# Impact of neoadjuvant androgen deprivation therapy on magnetic resonance imaging features in prostate cancer before radiotherapy

**DOI:** 10.1016/j.phro.2021.01.004

**Published:** 2021-02-24

**Authors:** Ulrika Björeland, Tufve Nyholm, Joakim Jonsson, Mikael Skorpil, Lennart Blomqvist, Sara Strandberg, Katrine Riklund, Lars Beckman, Camilla Thellenberg-Karlsson

**Affiliations:** a Department of Radiation Sciences, Umeå University, Umeå, Sweden; b Department of Molecular Medicine and Surgery, Karolinska Institutet, Stockholm, Sweden

**Keywords:** Prostate, mpMRI, Androgen deprivation, Texture, GLCM

## Abstract

**Background and purpose:**

In locally advanced prostate cancer (PC), androgen deprivation therapy (ADT) in combination with whole prostate radiotherapy (RT) is the standard treatment. ADT affects the prostate as well as the tumour on multiparametric magnetic resonance imaging (MRI) with decreased PC conspicuity and impaired localisation of the prostate lesion. Image texture analysis has been suggested to be of aid in separating tumour from normal tissue. The aim of the study was to investigate the impact of ADT on baseline defined MRI features in prostate cancer with the goal to investigate if it might be of use in radiotherapy planning.

**Materials and methods:**

Fifty PC patients were included. Multiparametric MRI was performed before, and three months after ADT. At baseline, a tumour volume was delineated on apparent diffusion coefficient (ADC) maps with suspected tumour content and a reference volume in normal prostatic tissue. These volumes were transferred to MRIs after ADT and were analysed with first-order -and invariant Haralick -features.

**Results:**

At baseline, the median value and several of the invariant Haralick features of ADC, showed a significant difference between tumour and reference volumes. After ADT, only ADC median value could significantly differentiate the two volumes.

**Conclusions:**

Invariant Haralick -features could not distinguish between baseline MRI defined PC and normal tissue after ADT. First-order median value remained significantly different in tumour and reference volumes after ADT, but the difference was less pronounced than before ADT.

## 1 Introduction

Prostate cancer is one of the most common cancers among men. For locally advanced prostate cancer, androgen deprivation therapy (ADT) is a standard treatment in combination with whole prostate radiotherapy (RT). ADT reduces prostate volume [1], [2], [3] and functions as a radiosensitiser [4]. A well-defined and precise RT is a key to successful treatment. Studies have shown that local recurrences often appear at or close to a dominant prostatic lesion [5], [6]. It has, therefore, been suggested that tumour volumes within the prostate should be targeted with dose escalation, boosting, delivered with a dose-painting technique [7].

To characterise prostate cancer with magnetic resonance imaging (MRI), American College of Radiology, has introduced: Prostate Imaging-Reporting and Data System (PI-RADS) v. 2.1 [8]. PI-RADS describes how to localise and grade prostate cancer depending on site, location, and extent. PI-RADS includes T2-weighted MRI (T2w), diffusion-weighted MRI (DWI), and T1-weighted MRI (T1w) + Gadolinium (Gd) dynamic contrast-enhanced (DCE) MRI. T2w illustrates excellent soft-tissue contrast. DWI measures water mobility in the tissue and correlates with cellularity and cell membrane integrity [9]. DWI is quantified using an apparent diffusion coefficient (ADC). Highly cellular areas such as tumours show restricted diffusion and appear as hypointense regions in the ADC map [9], [10]. Tumours differ from normal tissue in many aspects, including having a higher proportion of leaking capillaries. DCE measures blood vessel wall leakage from intravascular to extravascular space continuously by imaging the inflow of the injected Gd in the tissue [11] and can be quantified and visualised as a kinetic parameter, Ktrans [11].

ADT affects the tumour and results in decreased prostate cancer conspicuity on MRI [1], [2], [3], [12], [13]. This challenges the use of MRI, after neoadjuvant hormonal therapy, for RT target delineation. There is a growing interest in the use of advanced imaging for dose prescription and planning [14]. For MRI, image texture analysis has been suggested as a supporting tool to separate tumour from normal tissue [15], even in cases with low contrast between tumour and surrounding tissue [13]. Texture features for prostate cancer [16], [17], [18], [19] can be derived using grey-level co-occurrence matrices (GLCM) with the aim to separate or classify different tissue types. To do so, different statistical features can be extracted from GLCM such as Haralick features [20], thoroughly described and applied in several studies, both in its original form [13], [21], and in an invariant form independent of the number of grey-levels [22], [23]. Evaluating prostate cancer MRI-image data with the GLCM approach may improve tumour localisation after ADT and might therefore be of use in RT planning.

The aim of the study was to investigate the impact of ADT on baseline defined MRI features in prostate cancer. We hypothesised that significant differences exist in first-order and GLCM-based second-order statistics both before and after ADT, between tumour and reference volumes.

## 2 Materials and Methods

### 2.1 Study design and imaging

The present evaluation was a part of the phase *2 PARAPLY study;* ***(****NCT01962324) High-Risk Prostate Cancer Treated With Dose-escalated Simultaneous Integrated Boost to Prostate and Lymph Node GTV.* In this study, we evaluated two sets of MRI scans from the PARAPLY study. Baseline MRI was performed before ADT and the second MRI three months after ADT, before the start of RT. Patients were recruited consecutively during consultation at the Cancer Centre at Umeå University Hospital. Written informed consent was obtained from all participants. The study was approved by the regional ethics review board (approval number: 2013/154-31). In total, 84 consecutive patients were included in the PARAPLY study. After exclusion according to criteria listed in supplementary material Fig. S1, 50 patients remained. The MRI system used was a 3T PET/MRI (Signa, General Electric, Waukesha, WI, USA), for scanning parameters see Table S1 in supplementary materials. All scans were performed with a flat tabletop and with similar fixation equipment as during RT. Prior to MRI, patients were injected with 1 mg Glucagon subcutaneously to reduce bowel motion. The quantifiable parameters ADC-, Ktrans- and were calculated in MICE toolkit (NONPI Medical AB, Umeå, Sweden).

**Fig. 1:**
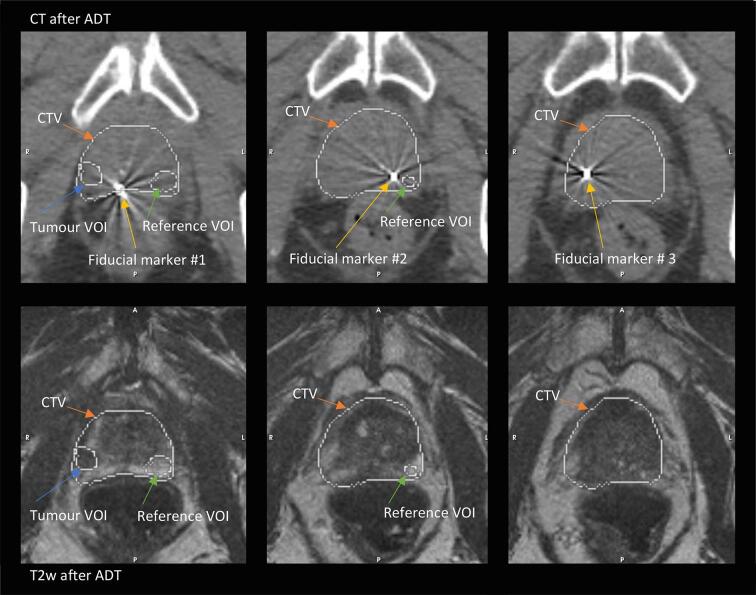
Fiducial markers in CT (top row) and T2w MRI (bottom row) after ADT in three different locations. Fiducial markers are inserted after ADT. VOIs indicate the tumour VOI and reference VOI registered with r + DIR techniques from ADC at baseline.

All patients were treated with neoadjuvant ADT (LHRH-analog) three months before RT start. Fiducial markers (Civco, Standard Gold Soft Tissue Markers, size: 3 mm × 1.2 mm) were placed in the prostate after ADT for RT guidance. See Fig. 1 for fiducial markers in computed tomography (CT) and the relation to the volumes of interests defined in section 2.2. All patients received external beam RT (EBRT) of 2.2 Gy to 77 Gy to the prostate in 35 fractions, pelvic nodes and seminal vesicles 1.6 Gy to 56 Gy in 35 fractions. A simultaneous integrated boost (SIB) to 84 Gy (fractional dose 2.4 Gy) was delivered to the dominant intraprostatic lesion if visualised.

### 2.2 Definitions, VOI identification, and registration process

**Fig. 2:**
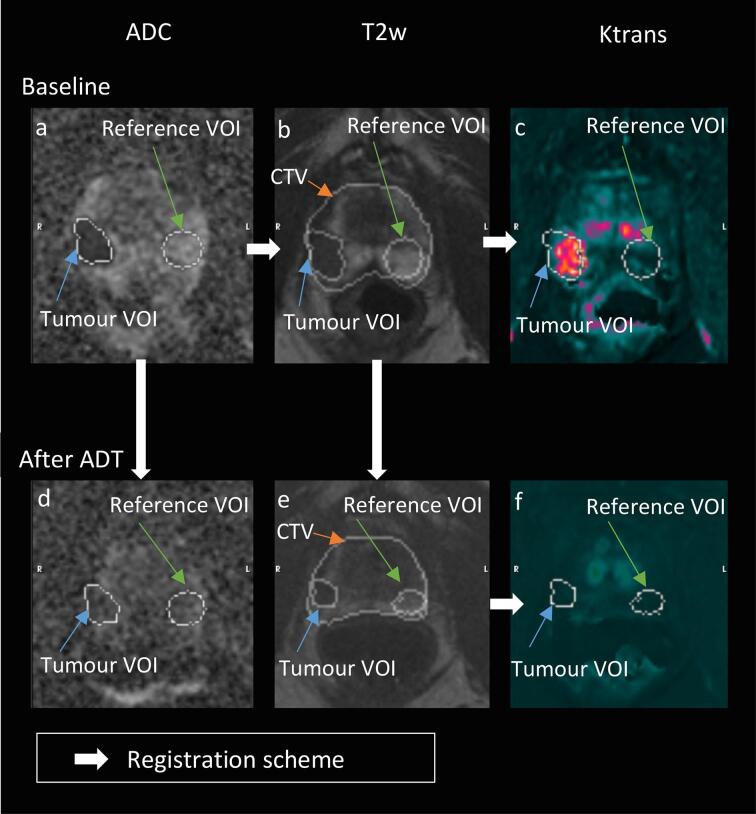
Multiparametric MRI (ADC, T2w and Ktrans from DCE MRI) from a patient in the study. a-c are baseline, and d-f are post-ADT. White arrows indicate registration order. PSA at baseline: 48 ng/ml, PSA post ADT: 0.1 ng/ml. a) ADC at baseline: Tumour VOI = 0.5 × 10^−3^ mm^2^/s, Reference VOI = 1.5 × 10^−3^ mm^2^/s. b) T2w at baseline, volumes: CTV = 55 cm^3^, Tumour VOI = 2.9 cm^3^, Reference VOI = 5.7 cm^3^. c) Ktrans at baseline: Tumour VOI = 0.11 min^−1^, Reference VOI = 0.02 min^−1^. d) ADC after ADT: Tumour VOI = 1.0 × 10^−3^ mm^2^/s, Reference VOI = 1.0 × 10^−3^ mm^2^/s. e) T2w after ADT, volumes: CTV = 35 cm^3^, Tumour VOI = 1.4 cm^3^, Reference VOI = 4.3 cm^3^. f) Ktrans after ADT: Tumour VOI = 0.02 min^−1^, Reference VOI = 0.03 min^−1^.

For each baseline MRI, an ADC volume of interest (VOI) where the signal indicated suspected prostate cancer, was outlined by two radiologists in consensus (LB, MS with 23 and 15 years of experience in MRI, respectively). This VOI was denoted as tumour and indicated a potential volume for RT escalation. Similarly, a prostate reference VOI was outlined in ADC at the corresponding contralateral side from the tumour by the main author (UB). Tumour- and reference- VOIs were then transferred to all other images with a combination of rigid and deformable image registration (r + DIR) techniques. Although radiologist outlined only the tumour VOI in ADC, we have chosen to name the transferred tumour VOI for tumour VOI in all other imaging types. The transferred reference VOI is called reference VOI in all other image types, see supplementary materials Table S2 for details in VOI definitions, and Fig. 2 for images. In the delineation process, all baseline MR images, as described in 2.1, were available to the radiologists.

All image registrations were performed in Oncentra or Elastix [24] interfaced through MICE-Toolkit. In the registration process, the underlying b = 200 s/mm^2^ was used instead of ADC due to better image contrast for image registration. Tumour and reference VOIs were transferred automatically with r + DIR techniques, from baseline diffusion-weighted image b = 200 s/mm^2^ to the examination three months later, after ADT. The r + DIR technique compensated for prostate motion [25] and deformation, but also image distortions. After visual inspection of the registrations and VOIs, 16 patients had an individualised optimisation of the Elastix parameters. The need for individualisation was mainly due to rectal gas and the following gas-induced susceptibility artefacts on DWI. The Elastix parameter files for the most common rigid and deformable registrations are shown in Appendix A. All images were resampled to ADC resolution.

The clinical target volume (CTV) was delineated, after ADT, in the treatment planning system Oncentra by oncologists. CTV was outlined on T2w with CT as an overlay, registered together with a rigid registration based on three fiducial markers in the prostate. The registration process minimises the quadratic sum between the same fiducial in the MRI and CT image. CTV at baseline was derived from registrations in MICE.

### 2.3 First- and second-order features

The MRIs included in the feature evaluation was ADC and Ktrans performed before and after ADT. The First-order features, Mean, Median, Max, Standard deviation, Skewness, Kurtosis, 5%-percentile, and 95%-percentile were computed in MICE for the VOIs defined in 2.2, and the median will be scrutinised further. The median value will be more insensitive, than, for example, mean value, if the fiducials are implanted in tumour VOI. The second-order features, GLCMs, for the VOIs defined in 2.2, were computed in MICE-Toolkit, with 32 bins, in four directions (horizontal, vertical and two diagonals) and separate for each slice. The GLCMs for the four directions were then combined and analysed as mean GLCM for the different VOI types. Invariant Haralick texture features [22], [23], [26] were calculated from the mean GLCM. See Tables S3 and S4 in supplementary materials, for all computed features.

### 2.4 Image statistics and evaluation

Relative PSA- and CTV volume- changes were monitored for each patient, and the normalised mean value was calculated. Also, VOI changes were calculated, see Appendix B for equations (Eqs. (B1)–(B6)). For all image types and time points, the tumour and reference VOIs were analysed with first-order statistics and with invariant GLCM Haralick textural features analysis. VOI differences were analysed with Wilcoxon Signed Ranks Test for dependent samples, for ADC and Ktrans, with a Bonferroni correction yielding a significance level of p < 0.0019. Spearman's rank correlation coefficient was used for correlation tests between features with a significance level of p < 0.01. All statistical analyses were performed using SPSS v.25.

## 3 Results

Relative PSA was decreased for all included patients after ADT. In the volumetric evaluation on T2w images, 48 patients were included, and 42 patients had a reduced relative CTV. For 44 patients, the relative tumour volume was reduced, and 43 patients had reduced reference volume, see Table 1.

**Table 1:** PSA and volumes.

	Relative Mean: reduction after ADT	Reduction in # % of the patients	Baseline mean	After ADT mean
PSA (ng/ml)	−97% ± 3%	100%	37.9	0.9
CTV (cm^3^)	−17% ± 30%	88%	56.2	44.1
Tumour volume (cm^3^)	−24% ± 16%	92%	5.8	4.3
Reference volume (cm^3^)	−27% ± 17%	90%	2.3	1.7

### 3.1 ADT influence on features

A significant difference in ADC between tumour at baseline and tumour after ADT was found for the first-order features for Mean, Median, Max, Standard deviation, and 95%-percentile. The same pattern was seen for Ktrans, with the addition of the 5%-percentile feature. For the reference VOI in ADC, a significant difference was found between baseline and after ADT, for the features Mean, Median, Max, 5%-percentile, and 95%-percentile. The same pattern was seen for the reference VOI in Ktrans with the addition of the Standard deviation feature. The largest significant VOI change was found for tumour VOIs in Ktrans. For second-order GLCM features, significant differences were only found in ADC for tumour at baseline vs after ADT, where 9 out of 20 features showed a significant difference between the two VOIs. No significant differences in tumour or reference features were noted for Ktrans VOIs between baseline and after ADT. For details, see supplementary materials Table S3.

### 3.2 Differences between tumour and reference VOIs

Significant differences were seen in ADC between tumour and reference at baseline for the first-order features; Mean, Median, Max, Standard deviation, 5%-percentile, and 95%-percentile. The same pattern was seen in Ktrans, except for 5%-percentile. After ADT, only ADC Mean, Median, and 5%-percentile showed a significant difference between tumour and reference. For second-order GLCM features, significant differences were only found in ADC between tumour and reference at baseline, where 7 out of 20 features showed a significant difference. In Ktrans no significant differences in baseline or after ADT features were noted between tumour and reference VOIs. The largest significant VOI change was found for baseline VOIs in ADC. For details, see supplementary materials Table S4.

### 3.3 First-order feature: Median

**Fig. 3:**
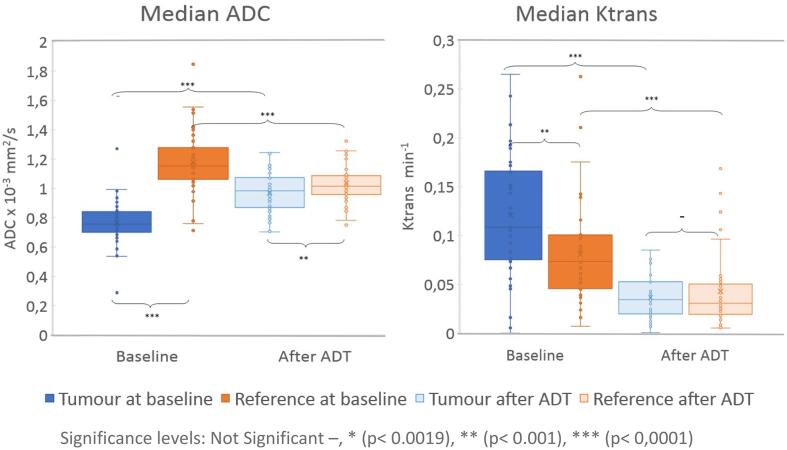
Median values in box plot representation before and after ADT for different imaging occasions and different modalities. The result from comparison between different VOIs by Wilcoxon Signed Rank test with significance levels: Not Significant –, * (p < 0.0019), ** (p < 0.001), *** (p < 0.0001). Median ADC: Tumour VOI at baseline **=** 0.8 ± 0.1 × 10^−3^ mm^2^/s, Tumour VOI after ADT = 0.9 ± 0.1 × 10^−3^ mm^2^/s, Reference VOI at baseline = 1.2 ± 0.2 × 10^−3^ mm^2^/s and Reference VOI after ADT = 1.0 ± 0.1 × 10^−^3 mm^2^/s. Median Ktrans: Tumour VOI at baseline VOI = 0.12 ± 0.07 min^−1^, Tumour VOI after ADT = 0.04 ± 0.02 min^−1^, Reference VOI at baseline = 0.08 ± 0.05 min^−1^ and Reference VOI after ADT = 0.04 ± 0.04 min^−1^.

A significant difference was found between baseline and after ADT for all median VOIs with the largest VOI change for the tumour VOI in Ktrans, see Tables S3 and S4. The median ADC value can also differentiate between reference and tumour VOI in both baseline and after ADT. For Ktrans images, the tumour VOI and the reference VOI was only significantly different at baseline. The ADC in the tumour VOI is rising from baseline to after ADT, and the reference VOI becomes lower after ADT. In baseline ADC the tumour VOI has a lower ADC than the reference, and after ADT this is still valid, but the difference is less pronounced, see Fig. 3. In baseline Ktrans, the tumour VOI has a higher Ktrans than the reference. Both tumour VOI and reference VOI in Ktrans becomes lower after ADT than in baseline, but the differences between tumour VOI and reference VOI is less after ADT, see Fig. 3.

Correlations between PSA and median tumour and reference VOIs in ADC and Ktrans images are summarised in Table 2. The PSA at baseline was not significantly correlated with median ADC or Ktrans values in neither tumour VOI or reference VOI at baseline nor after ADT. However, we found significant correlations between ADC and Ktrans VOIs. The strongest correlation was found in ADC after ADT, between tumour and reference. For ADC also, a significant correlation was seen between reference at baseline and reference after ADT. For Ktrans, there was a significant correlation between tumour and reference after ADT as well as for tumour and reference at baseline. A significant correlation between ADC and Ktrans was found on two occasions. First between tumour in ADC after ADT, and reference Ktrans after ADT, second between reference in ADC after ADT, and reference in Ktrans at baseline.

**Table 2:** Spearman’s rank correlation coefficient for correlation tests between features and PSA with a significance level of p < 0.01.

Correlation test ρ-values with p < 0.01			ADC Median				Ktrans Median			
		PSA at Baseline	Tumour at Baseline	Reference at Baseline	Tumour after ADT	Reference after ADT	Tumour at Baseline	Reference at Baseline	Tumour after ADT	Reference after ADT
	PSA at Baseline	1,00								

**ADC Median**	Tumour at Baseline		1,00							
	Reference at Baseline			1,00		0,48				
	Tumour after ADT				1,00	0,62		0,49		
	Reference after ADT			0,48	0,62	1,00		0,55		

**Ktrans Median**	Tumour at Baseline						1,00	0,55		
	Reference at Baseline				0,49	0,55	0,55	1,00		
	Tumour after ADT								1,00	0,60
	Reference after ADT								0,60	1,00

## 4 Discussion

The rationale for this study was to improve imaging-based radiotherapy planning in prostate cancer, by evaluating the impact of ADT on MRI features. One of the challenges in radiotherapy planning in prostate cancer is to identify the tumour on imaging post-ADT. We hypothesised that significant differences could be identified in both first- and GLCM-based second-order statistics, both before and after ADT, between tumour and reference VOIs. However, this was not the case. Especially after ADT, GLCM did not prove as robust as first-order features for distinguishing between tumour and reference VOI, neither with ADC nor Ktrans.

We chose to evaluate only quantifiable parameters in MRI performed with a standardised patient setup (including coils, scanner, and imaging parameters) to maximise reproducibility. The strength of our study is the prospective approach, a large number of patients and performing serial MRI in a standardised manner in the same patient population before and after ADT. The use of registrations to transfer the two types of VOIs from ADC at baseline to subsequent images could be seen as a weakness as it introduces registration uncertainties. The reason why we used registrations to transfer the VOIs between timepoints was because of the difficulties in identifying equivalent volumes manually due to the change in image contrast after ADT. No biopsies were taken from the different VOIs, so we cannot specify the pathological content in the volumes, and this is a weakness. Thus, only the VOIs in ADC at baseline were delineated manually by a radiologist.

We have found only three other studies monitoring similar patients before and after ADT with MRI (ADC [2] and ADC + DCE [1], [3]). In all three studies, as in ours, the ADC values in normal tissue decreased after ADT and the decrease of the prostate volume is also constant in all studies. In the two other studies that investigated Ktrans [1], [3] there was a significant decrease in tumorous tissue but not in the normal tissue, again in line with our results. The only differing result is that of ADC in the tumours after ADT where Barret et al. found a small decrease whilst our study and those by Kim et al. and Hötker et al. demonstrated an increase.

Comparison between different studies is not an easy task. Despite similarities in patient demographics and imaging protocols, differences in complex evaluation procedures pose a problem. Nevertheless, some of the results mentioned above can be applied to support our findings. In our study, the median ADC value in the baseline defined tumour VOI increased significantly after ADT. At the same time, the normal tissue decreased significantly after ADT, and the same result was found by Kim et al. [2] and Hötker et al. [3]. These phenomena can also be observed by visual inspection, see Fig. 2a,d. ADT is known to cause devascularisation in both tumorous and normal prostatic tissue [27]. Mean tumour Ktrans decreased significantly after ADT in our study, as well as in the studies by Barret et al. [1] and Hötker et al. [3]. In addition, after ADT, we found a significant difference in mean Ktrans also for normal reference tissue.

Significant correlations were found between tumour and reference VOIs for ADC and Ktrans, with the strongest correlation in ADC after ADT. This suggests that ADT influences ADC images of tumorous prostatic tissue by reducing the difference in signal intensity compared to normal tissue. Also, for ADC, there was a significant correlation in the reference VOI at baseline and after ADT indicating that ADT does not influence normal tissue ADC to the same extent as in tumorous tissue ADC.

We showed prostate (CTV) shrinkage and a reduced PSA after ADT, so did Barret et al. [1], Kim et al. [2], and Hötker et al. [3]. Both CTV and VOIs were reduced after ADT. Nevertheless, we observed relatively smaller volume reductions after ADT, which may be explained by the different VOI-outlining methodologies. In our study, as well in Barret et al. [1], median tumour ADC at baseline did not correlate with baseline PSA. This indicates that PSA and ADC measure different biological events that are not linked directly.

Grey-level patterns from radiological images, used in radiomics, have been evaluated before [18], [19], [28], and GLCM and Haralick [13], [22], [23], [26] features are commonly used. Daniel et al. [13] studied first order- and the GLCM- features before and after ADT. T2w and ADC features for tumours in the peripheral zone or central gland were compared. Just as Daniel et al., we observed a significant difference in mean ADC before and after ADT. However, our data do not indicate that GLCM features are superior to first-order features for ADC, such as mean ADC, as found by Daniel et al., but rather the opposite. One explanation of the differences in outcome might be that Daniel et al. [13] evaluated two different patient groups, with and without ADT. In contrast, we have assessed the same patients before and after ADT.

To conclude, we evaluated the impact of ADT on MRI texture analysis features and the ability to differentiate the baseline defined tumour- from a non-tumour reference-VOI pre and post ADT. Of first-order texture features, only ADC defined texture features were significantly different between tumour and normal tissue after ADT, which is in line with the visual increase as well as a noted median increase in ADC compared to reference tissues post-ADT. None of the GLCM- texture features could differentiate between baseline defined tumour VOI and normal tissue measured after ADT and therefore not be of use in radiotherapy planning.

## Declaration of Competing Interest

The authors declare the following financial interests/personal relationships which may be considered as potential competing interests: Ulrika Björeland, Lars Beckman, Sara Strandberg, Mikael Skorpil, Katrin Riklund and Camilla Thellenberg-Karlsson declare that they have no conflict of interest. Joakim Jonsson and Tufve Nyholm are shareholders in NONPI Medical AB. Lennart Blomqvist is cofounder of Collective Minds Radiology (www.cmrad.com).
