# Supplementary material for: Impact of neoadjuvant androgen deprivation therapy on magnetic resonance imaging features in prostate cancer before radiotherapy
Source: Phys Imaging Radiat Oncol. 2021 Feb 24;17:117–23. doi: 10.1016/j.phro.2021.01.004 (PMC8058024; doi:10.1016/j.phro.2021.01.004)
Supplement: Supplementary data [file mmc1.pdf]

Appendix A: General elastix parameters

Rigid registration:

The most common parameters altered in the rigid registration were Number Of Histogram Bins (16, 32, 64, 128) and Number Of Resolutions (2-4).

```
(FixedInternalImagePixelType "float")

(MovingInternalImagePixelType "float")

(FixedImageDimension 3)

(MovingImageDimension 3)

// **************** Main Components **************************

(Registration "MultiResolutionRegistration")

(Interpolator "LinearInterpolator")

(ResampleInterpolator "FinalBSplineInterpolator")

(Resampler "DefaultResampler")

(FixedImagePyramid "FixedSmoothingImagePyramid")

(MovingImagePyramid "MovingSmoothingImagePyramid")

(Optimizer "AdaptiveStochasticGradientDescent")

(Transform "EulerTransform")

(Metric "AdvancedMattesMutualInformation")

// ***************** Transformation **************************

(AutomaticTransformInitialization "true")

(AutomaticTransformInitializationMethod "GeometricalCenter")

(HowToCombineTransforms "Compose")

// ******************* Similarity measure *********************

(NumberOfHistogramBins 64 128)

(ErodeMask "false")

// ******************** Multiresolution **********************

(NumberOfResolutions 2)

// ******************* Optimizer ****************************

(MaximumNumberOfSamplingAttempts 10.000000)

(RequiredRatioOfValidSamples 0.25)

(MaximumStepLength 4.0 2.0 1.0)

// **************** Image sampling **********************

(NumberOfSpatialSamples 2048)

(NewSamplesEveryIteration "true")

(ImageSampler "RandomCoordinate")

// ************* Interpolation and Resampling ****************

(FinalBSplineInterpolationOrder 3)
```

```
(DefaultPixelValue 0)
(ResultImagePixelType "float")
```

Deformable registration:

```
(FixedInternalImagePixelType "float")
(MovingInternalImagePixelType "float")
(FixedImageDimension 3)
(MovingImageDimension 3)
(UseDirectionCosines "true")
// **************** Main Components **************************
(Registration "MultiMetricMultiResolutionRegistration")
(Interpolator "LinearInterpolator")
(ResampleInterpolator "FinalBSplineInterpolator")
(Resampler "DefaultResampler")
(FixedImagePyramid "FixedSmoothingImagePyramid")
(MovingImagePyramid "MovingSmoothingImagePyramid")
(Optimizer "AdaptiveStochasticGradientDescent")
(Transform "BSplineTransform")
(Metric "AdvancedMattesMutualInformation" "TransformBendingEnergyPenalty")
(Metric0Weight 0.8)
(Metric1Weight 0.2)
// ******************** Multiresolution **********************
(NumberOfResolutions 4)
// ***************** Transformation **************************
(FinalGridSpacingInPhysicalUnits 25)
(HowToCombineTransforms "Compose")
// ******************* Similarity measure *********************
(NumberOfHistogramBins 64)
(ErodeFixedMask "false")
(ErodeMovingMask "false")
// ******************* Optimizer ****************************
(MaximumNumberOfIterations 1000)
(MaximumNumberOfSamplingAttempts 8)
// **************** Image sampling **********************
(NumberOfSpatialSamples 2048)
(NewSamplesEveryIteration "true")
(ImageSampler "RandomCoordinate")
// ************* Interpolation and Resampling ****************
```

(FinalBSplineInterpolationOrder 3)

(DefaultPixelValue 0)

(ResultImagePixelType "float")

Appendix B: Equations

$$Mean\ PSA = \frac{PSA\ at\ baseline - PSA\ after\ ADT}{PSA\ at\ baseline}$$ Eq.B1

The volume changes were calculated in T2w for every patient, and then the mean value was calculated:

$$Mean\ Volume\ CTV = \frac{CTV\ at\ baseline - CTV\ after\ ADT}{CTV\ at\ baseline}$$ Eq.B2

$$Mean\ tumour\ Volume\ = \frac{Tumour\ VOI\ at\ baseline - Tumour\ VOI\ after\ ADT}{Tumour\ VOI\ at\ baseline}$$ Eq.B3

$$Mean\ reference\ Volume\ = \frac{Reference\ VOI\ at\ baseline - Reference\ VOI\ after\ ADT}{Reference\ VOI\ at\ baseline}$$ Eq.B4

$VOI\ change\ \_ADT: \frac{Tumour\ VOI\ after\ ADT}{Tumour\ VOI\ at\ Baseline}$ repeated for reference VOI. Applied to ADC and Ktrans Eq.B5

$VOI\ change\_Type: \frac{Reference\ VOI\ at\ baseline}{Tumour\ VOI\ at\ baseline}$ repeated for after ADT. Applied to ADC and Ktrans Eq.B6

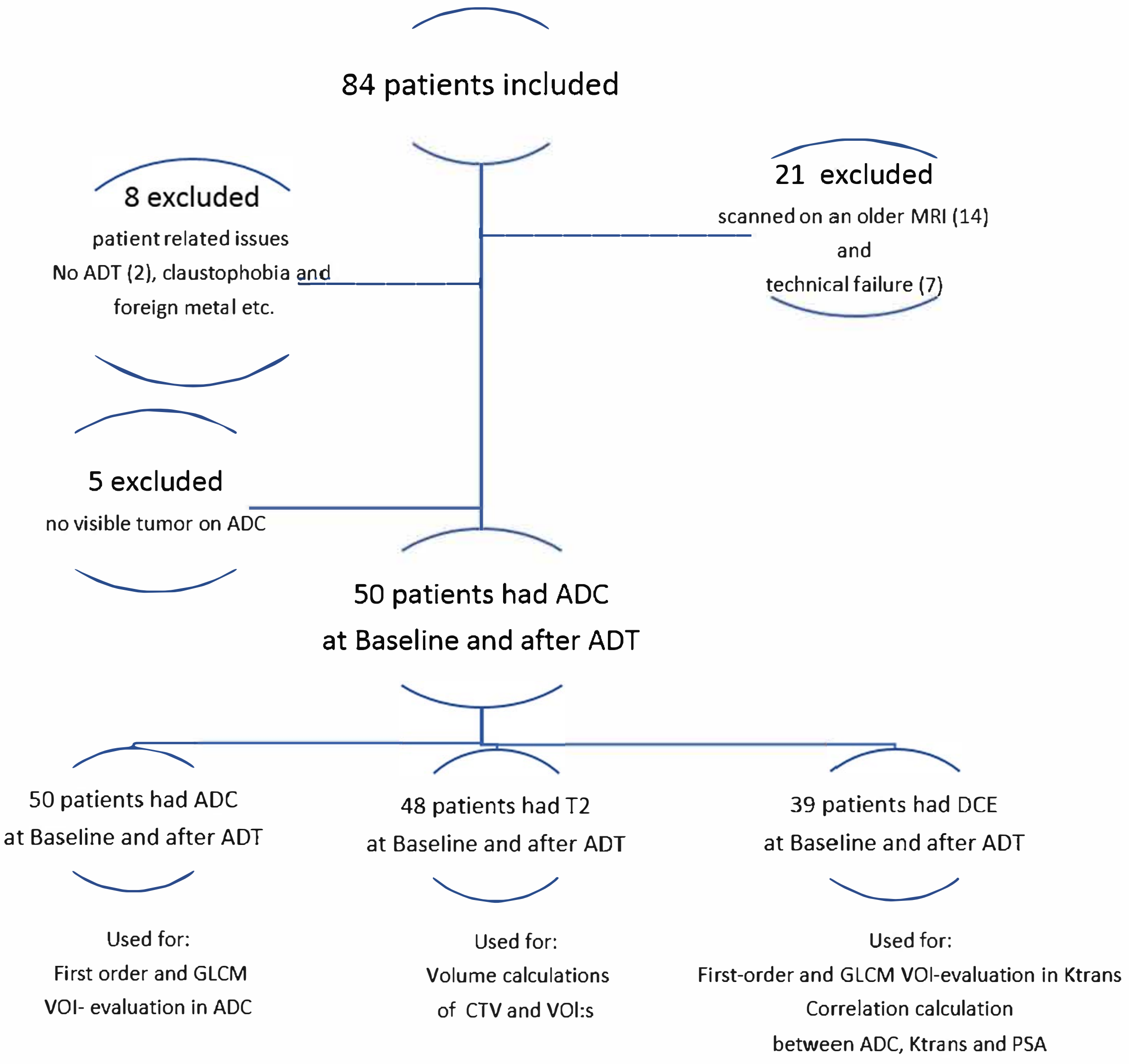

Fig. S1. Overview of included/excluded patients and evaluation for each group.

Table S1. Imaging parameters.

Images	Parameters
T2w	T2-Fast Recovery Fast Spin Echo (FRFSE),<br>TE=97.6 ms, TR=9873 ms, matrix: 512x512x52, voxel: 0.5mm x 0.5mm x 2mm, no gap
Diffusion-weighted	DWI-Field of view Optimized and Constrained Undistorted Single shot (FOCUS),<br>TE=69.4 ms, TR=3500 ms, matrix: 256x256x16, voxel: 0.9 mm x0.9 mm x 4mm, 4 average, b-values: 200 s/mm^2 and 800 s/mm^2
T1w DCE	T1-Fast-spoiled-gradient-recalled-echo (SPGR)+Gd,<br>TE=1.88 ms, TR=4.36 ms, matrix: 256x256x12, voxel: 1mm x 1mm x 5mm , mean temporal resolution 11.3 s, gadoterate meglumine (Dotarem) 0.2 ml/kg

Table S2. VOI definitions.

Image type	Baseline		After ADT	
	**Tumour VOI**	**Reference VOI**	**Tumour VOI**	**Reference VOI**
ADC	VOI encloses the volume were signal indicated suspected tumour content	VOI outlined at the corresponding contralateral side from the tumour	Used r+DIR to transfer Tumour VOI from ADC Baseline	Used r+DIR to transfer Reference VOI from ADC Baseline
T2w	Used r+DIR to transfer Tumour VOI from ADC Baseline	Used r+DIR to transfer Reference VOI from ADC Baseline	Used r+DIR to transfer Tumour VOI from T2 Baseline	Used r+DIR to transfer Reference VOI from T2 Baseline
Ktrans	Used r+DIR to transfer Reference VOI from T2 Baseline	Used r+DIR to transfer Reference VOI from T2 Baseline	Used r+DIR to transfer Tumour VOI from T2 after ADT	Used r+DIR to transfer Reference VOI from T2 after ADT

Table S3. First-order- and second order Invariant GLCM -features for ADC and Ktrans.

ADT influence on tumor VOI and reference VOI in the prostate for all patients in the respective group. Significant difference by Wilcoxon-test, $p < 0,0019$. Significance is coded in green for $p < 0,0019$ and gray for $p > 0,0019$.

VOI change_ADT is represented for each feature:

If VOI change_ADT < 1: VOI after ADT < VOI at Baseline

If VOI change_ADT > 1: VOI after ADT > VOI at Baseline

If VOI change_ADT = 1: VOI after ADT = VOI at Baseline

Wilcoxon Signed Rank test<br>p< 0.0019 is significant<br>p > 0,05<br>p < 0,05<br>p < 0,01<br>p < 0,0019<br>p < 0,001<br>p < 0,0001	ADC			
	Tumour VOI after ADT vs Tumour VOI at Baseline	Reference VOI after ADT vs Reference VOI at Baseline	Tumour VOI after ADT vs Tumour VOI at Baseline	Reference VOI after ADT vs Reference VOI at Baseline
	numbers: VOI change_ADT	numbers: VOI change_ADT	numbers: VOI change_ADT	numbers: VOI change_ADT
**First order features**				
Mean	1,31	0,89	0,48	0,73
Median	1,32	0,89	0,60	0,73
Max	1,31	0,93	0,45	0,77
Standard Deviation	1,45	1,04	0,45	0,78
Skewness (Fisher-Pearson)	7,59	-7,04	1,41	0,69
Kurtosis	1,02	1,07	1,57	1,24
5% Percentile	1,16	0,82	0,26	0,90
95% Percentile	1,33	0,92	0,45	0,74
**Second order GLCM Invariant features**				
Contrast	0,86	1,01	1,43	1,30
Inverse Difference	1,01	1,00	0,99	0,99
Entropy	1,04	1,01	1,05	1,06
Energy	1,09	1,05	1,55	1,25
MaximumProbability	1,07	1,07	1,43	1,22
Correlation	1,17	1,00	1,00	1,00
Sum Of Squares Variance	1,11	0,98	1,33	1,35
Homogeneity	1,00	1,00	1,00	1,00
Dissimilarity	0,91	0,99	1,20	1,15
Sum Average	1,08	1,06	1,23	1,12
Sum Variance	1,19	0,99	1,35	1,37
SumEntropy	0,45	0,79	-0,38	3,71
Difference Variance	0,86	1,01	1,28	1,21
Difference Entropy	1,09	1,02	0,98	0,98
Information Measure Of Correlation1	1,48	1,01	1,68	1,59
Information Measure Of Correlation2	1,04	0,99	1,00	1,01
Auto correlation	1,19	1,13	1,62	1,34
Cluster Shade	-0,19	-10,10	0,45	1,24
Cluster Prominence	1,47	1,13	1,76	1,71
Difference Average	0,93	0,99	1,11	1,08

Table S4. First-order- and second order Invariant GLCM -features for ADC and Ktrans.
Ability to separate tumour VOI from reference VOI in the prostate at baseline or after ADT. Significant difference by Wilcoxon-test, p < 0,0019. Significance is coded in green for p< 0,0019 and gray for p> 0,0019.
VOI change_Type is represented for each feature:
If VOI change_Type < 1: Reference VOI < Tumour VOI
If VOI change_Type > 1: Reference VOI > Tumour VOI
If VOI change_Type = 1: Reference VOI = Tumour VOI

Wilcoxon Signed Rank test<br>p< 0.0019 is significant<br>p > 0,05<br>p < 0,05<br>p < 0,01<br>p < 0,0019<br>p < 0,001<br>p < 0,0001	ADC		Ktrans	
	Reference VOI at Baseline vs Tumour VOI at Baseline	Reference VOI after ADT vs Tumour VOI after ADT	Reference VOI at Baseline vs Tumour VOI at Baseline	Reference VOI after ADT vs Tumour VOI after ADT
	numbers: VOI change_Type	numbers: VOI change_Type	numbers: VOI change_Type	numbers: VOI change_Type
**First order features**				
Mean	1,57	1,07	1,01	0,73
Median	1,59	1,08	0,81	0,73
Max	1,38	0,98	0,81	0,77
Standard Deviation	1,37	0,98	0,78	0,78
Skewness (Fisher-Pearson)	1,63	-2,19	0,73	0,69
Kurtosis	0,95	1,00	1,24	1,25
5% Percentile	1,71	1,21	0,78	0,90
95% Percentile	1,49	1,03	0,88	0,74
**Second order GLCM Invariant features**				
Contrast	1,05	1,27	1,37	1,43
Inverse Difference	1,00	0,99	1,00	1,00
Entropy	1,02	1,01	1,01	1,07
Energy	1,01	1,02	1,21	1,37
MaximumProbability	1,04	1,10	1,14	1,35
Correlation	1,16	0,99	1,00	1,01
Sum Of Squares Variance	1,31	1,23	1,19	1,31
Homogeneity	1,00	1,00	1,00	1,00
Dissimilarity	1,01	1,11	1,16	1,20
Sum Average	1,08	1,06	1,09	1,12
Sum Variance	1,39	1,23	1,19	1,32
SumEntropy	-0,54	0,52	4,84	0,36
Difference Variance	1,03	1,24	1,28	1,29
Difference Entropy	1,02	0,96	0,99	1,00
Information Measure Of Correlation1	2,17	1,59	1,31	1,54
Information Measure Of Correlation2	1,10	1,05	1,01	1,02
Auto correlation	1,19	1,15	1,22	1,43
Cluster Shade	-2,44	12,22	0,22	1,90
Cluster Prominence	1,82	1,65	1,47	1,66
Difference Average	1,01	1,08	1,08	1,08
